# Groundwater quality assessment for drinking purposes: a case study in the Mekong Delta, Vietnam

**DOI:** 10.1038/s41598-023-31621-9

**Published:** 2023-03-16

**Authors:** Nguyen Thanh Giao, Huynh Thi Hong Nhien, Phan Kim Anh, Pumis Thuptimdang

**Affiliations:** 1 Department of Environmental Management, College of Environment and Natural Resources, Can Tho University, Can Tho, 900000 Vietnam; 2 Department of Chemistry, Faculty of Science, Chiang Mai University, Chiang Mai, 52000 Thailand; 3 Environmental Science Research Center, Faculty of Science, Chiang Mai University, Chiang Mai, 52000 Thailand

**Keywords:** Environmental sciences, Environmental impact

## Abstract

Groundwater serves as an important resource for people in the Mekong Delta, but its quality has been continuously declined from human activities. Current status of the groundwater quality needs to be evaluated for sustainable groundwater resource management. This study aimed to evaluate the groundwater quality for drinking purposes in the Mekong Delta, Vietnam, using multivariate statistical methods and integrated-weight water quality index. Data comprised 8 water quality parameters (pH, total hardness, nitrate (NO_3_^−^), iron (Fe), lead (Pb), mercury (Hg), arsenic (As), and coliforms) obtained from 64 observation wells in An Giang province, Dong Thap province, and Can Tho city, were analyzed by cluster analysis (CA), principal component analysis (PCA), and integrated-weight water quality index (IWQI). The results indicated that most parameters were within standards while excessive hardness and Fe contamination were found in some regions. More than 80% of samples were detected with serious coliform contamination. The CA results revealed that groundwater quality heavily depend on geological locations with 4 clusters of the sampling locations. Three principal components obtained from PCA could explain 77.2% of the groundwater quality variation. The IWQI values ranging from 4 to 2761 classified groundwater quality as excellent (53.1%), good (25%), poor (9.4%), very poor (4.7%), and undrinkable (7.8%), which were associated with coliform contamination. These findings have provided insights into the groundwater quality status in the region, which can benefit in developing a water protection strategy.

## Introduction

Groundwater is an integral freshwater source that serves different human needs in many parts of the world. For agricultural purposes, groundwater contributes to approximately 43% of worldwide water usage for irrigation^1^. Groundwater is also a major water source for household activities in many areas including the countries in Africa^2,3^ and Asia^4,5^. Despite its importance, the quantity and quality of groundwater resources are increasingly threatened by both natural and anthropogenic factors. Climate change has potential impacts on groundwater such as seawater intrusion and groundwater level decline^6,7^. Additionally, a rapid increase in water demand and contamination from anthropogenic pollutants such as fertilizer residue and untreated wastewater has worsened this water source^4,5,8^.

Similar to other areas, the groundwater in the Mekong Delta of Vietnam also faces the depletion of quantity and decline of quality from anthropogenic stressors. In recent decades, the river and canal systems in this region have been highly polluted by agricultural, aquacultural, domestic, and industrial wastes^9^. According to the study in Hau River^10^, one of the sources for this contamination is the untreated wastewater from rice intensification and domestic sewage pipes that was directly discharged into surface water bodies. This has forced people in the Mekong delta area to be more reliant on the groundwater source. According to Erban et al.^11^, the groundwater level in the region has annually declined with an average of approximately 0.3 m/year. Previous studies have reported various groundwater pollutants in the area including arsenic contamination in An Giang province^12,13^, ammonium contamination in Bac Lieu province^14^, and nitrate and chloride contamination in Soc Trang province^8^. Thus, it is imperative to continuously evaluate the groundwater quality in this region to ensure that its quality can serve human demands. Groundwater quality assessment is commonly conducted by comparing each key parameter with the drinking water standard limit. However, determining each water quality parameter independently can lead to the lack of the correlation among related parameters into the groundwater quality assessment.

For a better interpretation of the data, multivariate statistical approaches such as cluster analysis (CA) and principal component analysis (PCA) are necessary to provide more insights into water quality assessment. PCA can be applied to identify the key parameters correlated with the water quality and to determine potential pollution sources while CA is generally used to group sampling sites with the commonalities of water quality properties^14–16^. Furthermore, water quality index (WQI) has been widely employed to assess water quality and its suitability for drinking purposes^5,9,16,17^. One limitation of this method is the subjective weights of parameters that are generally defined by experts based on the importance of these corresponding parameters. This can lead to different weighting values for a single parameter, and any small changes will significantly affect the final WQI values. For this reason, an integrated-weight method that combines information entropy and Criteria Importance Through Inter-criteria Correlation (CRITIC) method has been applied to generate more accurate and objective WQI values and to overcome the subjective issue of the traditional approach by considering the conflict between each parameter’s correlation and the changes in data size and value^5,18,19^.

Therefore, this study aims to evaluate the groundwater quality in the Mekong Delta area by CA and PCA, and to determine the quality for drinking purposes by integrated-weight water quality index (IWQI) methods. The findings from this research not only demonstrate the importance of applying statistical techniques in groundwater quality assessment but also provide the drinking-quality status of the groundwater in the Mekong Delta region, which is significant to further groundwater resource management.

## Materials and methods

### Study area description

The study area includes two provinces (An Giang and Dong Thap) and one city (Can Tho city) of Vietnam, which are located within the Mekong Delta area. The study area is in tropical monsoon climate with two distinct seasons: the rainy season (May–October) and the dry season (November–April). In Mekong Delta, the groundwater exploitation mainly occurs in the Middle-Upper Pleistocene (qp_23_), Lower Pleistocene (qp_1_) and Upper Miocene (n_13_) layers. Each area description is provided below.

#### An Giang province

An Giang province is situated in the southwest of Vietnam (10° 12ʹ N to 10° 57ʹ N and 104° 46ʹ E to 105° 35ʹ E) and borders with Cambodia in the northwest. The east and northeast borders Dong Thap province while the south and southeast borders Can Tho city. With the area of 353,668.02 ha, the terrain of An Giang is divided into two typical types: 87% of plain terrain and 13% of mountainous areas. This has facilitated the area as an important agricultural center of Vietnam, particularly rice production. According to the land use map of An Giang province in 2018, the ratio of triple and double rice crops accounted for 46.6% and 24.7%, respectively^16^. Using surface water for dike irrigation systems has caused the deterioration of surface water and groundwater quality in the region^16,20^.

#### Can Tho city

The key economic region of Mekong Delta comprises not only An Giang province but also Can Tho city. With the area of 1439.2 km^2^, Can Tho city is the largest city in the Mekong Delta. The terrain is relatively flat and suitable for rice and fishery production. Surface water pollution is a current issue of the city where wastes from domestic and aquacultural activities and food processing factories are the main pollution sources; therefore, groundwater has become an important freshwater source to meet daily demands^21^.

#### Dong Thap province

Dong Thap province has a total area of 3384 km^2^, comprising 2602 km^2^ of agricultural land, 111 km^2^ of forest land, 257 km^2^ of special-use land, and 146 km^2^ of residential land. It has aquaculture and agriculture as the key sectors for its economic development.

### Groundwater sampling and analysis

**Figure 1:**
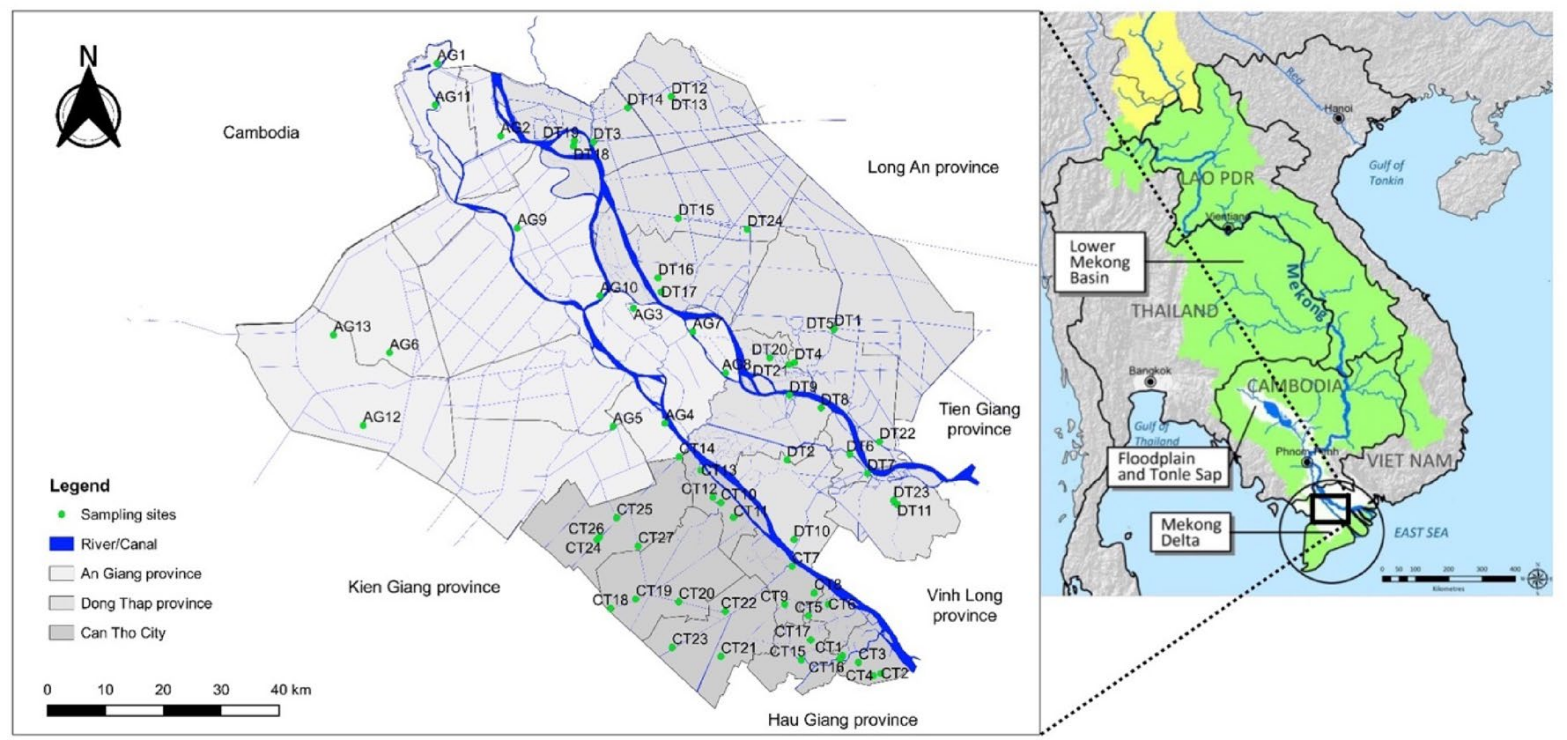
Map of groundwater sampling sites in the Mekong Delta of Vietnam (AG1-AG13 are sampling sites in An Giang province, DT1-DT24 are sampling sites in Dong Thap province, and CT1-CT27 are sampling sites in Can Tho city) located within the Mekong River basin. The map containing the sampling locations (left) was constructed by the software QGIS version 3.14 (https://qgis.org/en/site/forusers/download.html) licensed under GNU General Public License (CC BY-SA 3.0). The modification of the map was made directly in the OpenStreetMap data layer from the QGIS database. The Mekong River basin map (right) has been modified from Mekong River Commission (2020)^22^ licensed under CC BY 4.0.

Groundwater sampling collection and analysis were performed by the Center for Natural Resources and Environment Monitoring of An Giang province, Dong Thap province, and Can Tho city (Vietnam), in which the Ministry of Natural Resources and Environment (Vietnam) has granted a certificate of eligibility to operate environmental monitoring services. In 2019, groundwater samples were collected twice, one time in March and one time September, from 64 sampling sites along the Mekong River in Can Tho city (location denoted as CT1-CT27), An Giang province (location denoted as AG1–AG13) and Dong Thap province (location denoted as DT1-DT24). Figure 1 shows the sampling locations within study area of Mekong Delta, which is located within the Mekong River basin^22^. Water quality parameters including pH, total hardness, nitrate (NO_3_^−^), iron (Fe), lead (Pb), mercury (Hg), arsenic (As), and coliforms were measured. Only pH was measured on-site with a portable pH meter (model: SensION + pH1, Hach, USA) while other parameters were measured in the laboratory using the standard methods^23^, which include the cadmium reduction method for NO_3_^−^ (SMEWW 4500.NO_3_^−^E:2012), the flame and electrothermal atomic absorption spectrometric method (SMEWW 3112.B:2012 and SMEWW 3113.B:2012) for heavy metals (Fe, Pb, Hg and As), and the multiple-tube method (TCVN 6178-2:1996 (ISO 9308-2:1990 (E))) for coliforms.

### Multivariate statistical analysis

CA using the Ward’s linkage method was conducted to group groundwater sampling sites based on their similarity in terms of water quality. The clusters are formed based on the Euclidean distance calculated by (D_link_/D_max_) × 100, in which D_link_ is the linkage distance for an individual case and D_max_ is the maximum linkage distance. The number of statistically significant clusters is selected by considering the groups such that (D_link_/D_max_) × 100 < 60^24^. Z-score standardization was used to eliminate the effect of different units of water quality parameters. PCA was employed to extract key parameters that drive the variation of groundwater quality in this study. This method forms a new set of parameters known as principal component (PC)^9,13^. PC was selected based on its eigenvalue and percentage contribution to the total variation. Both CA and PCA were performed using the ORIGIN version 2019b (OriginLab, USA).

### Integrated-weight water quality index (IWQI)

#### Entropy-weighted method

The method of information entropy to calculate weighting parameters was from Shannon^25^. The calculation of entropy weights of parameters was conducted as follows:

Firstly, the initial data were arranged as Eq. (1):

1 $$X = \left[ {\begin{array}{*{20}c} {{\text{x}}_{11} } & \cdots & {{\text{x}}_{{{\text{1n}}}} } \\ \vdots & \ddots & \vdots \\ {{\text{x}}_{{{\text{m1}}}} } & \cdots & {{\text{x}}_{{{\text{mn}}}} } \\ \end{array} } \right]$$

where, m is the number of groundwater sampling sites (i = 1, 2, …, m), n is the number of water quality parameters (j = 1, 2, …, n).

Since the units of water quality parameters were different, the value of each parameter was standardized as expressed in Eq. (2):

2 $${\text{y}}_{{{\text{ij}}}} = \frac{{{\text{x}}_{{{\text{ij}}}} - \left( {{\text{x}}_{{{\text{ij}}}} } \right)_{\min } }}{{\left( {{\text{x}}_{{{\text{ij}}}} } \right)_{\max } - \left( {{\text{x}}_{{{\text{ij}}}} } \right)_{\min } }}$$

where, x_ij_ is the jth analyzed groundwater parameter of ith groundwater sampling site.

Then, the standardized data were organized as Eq. (3):

3 $${\text{Y}} = \left[ {\begin{array}{*{20}c} {{\text{y}}_{11} } & \cdots & {{\text{y}}_{{{\text{1n}}}} } \\ \vdots & \ddots & \vdots \\ {{\text{y}}_{{{\text{m1}}}} } & \cdots & {{\text{y}}_{{{\text{mn}}}} } \\ \end{array} } \right]$$

Information entropy (e_j_) was computed according to Eqs. (4) and (5):

4 $${\text{e}}_{{\text{j}}} = - \frac{1}{{{\text{lnm}}}}\mathop \sum \limits_{{{\text{i}} = 1}}^{{\text{m}}} {\text{f}}_{{{\text{ij}}}} {\text{lnf}}_{{{\text{ij}}}}$$

5 $${\text{f}}_{{{\text{ij}}}} = \frac{{{\text{y}}_{{{\text{ij}}}} + 10^{ - 4} }}{{\mathop \sum \nolimits_{{{\text{i}} = 1}}^{{\text{m}}} \left( {{\text{y}}_{{{\text{ij}}}} + 10^{ - 4} } \right)}}$$

where e_j_ is the information entropy of the jth parameter. 10^–4^ is used to ensure the formula is meaningful. m is the number of groundwater sampling sites.

Finally, the entropy weight (W_j1_) was calculated as follows:

6 $${\text{W}}_{{{\text{j1}}}} = \frac{{1 - {\text{e}}_{{\text{j}}} }}{{\mathop \sum \nolimits_{{{\text{j}} = 1}}^{{\text{n}}} \left( {1 - {\text{e}}_{{\text{j}}} } \right)}}$$

#### CRITIC-weighted method

The CRITIC-weighted method was applied according to Zhang et al.^5^, the weights (W_j2_) of water quality parameters were computed by Eqs. (7)–(9):

7 $${\text{W}}_{{{\text{j2}}}} = \frac{{{\text{s}}_{{\text{j}}} }}{{\mathop \sum \nolimits_{{{\text{j}} = 1}}^{{\text{m}}} {\text{s}}_{{\text{j}}} }}$$

8 $${\text{s}}_{{\text{j}}} = \delta_{{\text{j}}} \mathop \sum \limits_{{{\text{j}} = 1}}^{{\text{m}}} (1 - {\text{r}}_{{{\text{ij}}}} )$$

9 $${\text{r}}_{{{\text{ij}}}} = \frac{{\sum ({\text{y}}_{{{\text{ij}}}} - \overline{{{\text{y}}_{{{\text{ij}}}} }} )({\text{y}}_{{{\text{ij}}}}^{^{\prime}} - \overline{{{\text{y}}_{{{\text{ij}}}}^{^{\prime}} }} )}}{{\sqrt {\sum ({\text{y}}_{{{\text{ij}}}} - \overline{{{\text{y}}_{{{\text{ij}}}} }} )^{2} \sum ({\text{y}}_{{{\text{ij}}}}^{^{\prime}} - \overline{{{\text{y}}_{{{\text{ij}}}}^{^{\prime}} }} )^{2} } }}$$

where, W_j2_ represents the weight of the jth water quality parameter, s_j_ is the quantity of information calculated for the jth parameter, δ_j_ is the standard deviation of jth parameter, ${\text{r}}_{\text{ij}}$ is the correlation coefficient, $\overline{{\mathrm{y} }_{\text{ij}}}$ is the average value of the parameter ${\text{y}}_{\text{ij}}$, and ${\mathrm{y}}_{\mathrm{ij}}^{\mathrm{^{\prime}}}$ is the mean value of the parameter ${\mathrm{y}}_{\mathrm{ij}}^{\mathrm{^{\prime}}}$. The values of ${\text{y}}_{\text{ij}}$ and ${\mathrm{y}}_{\mathrm{ij}}^{\mathrm{^{\prime}}}$ were normalized by Eq. (2).

#### Integrated weight calculation

The integrated weights (W_j_) can be calculated by combining the entropy weights and CRITIC weights^5^, which are expressed in Eqs. (10)–(12):

10 $${\text{W}}_{{\text{j}}} = {\text{ pW}}_{{{\text{j1}}}} + (1 - {\text{p}}){\text{W}}_{{{\text{j2}}}}$$

11 $${\text{p }} = \mathop \sum \limits_{{{\text{j}} = 1}}^{{\text{m}}} \left[ {\left( {{\text{w}}_{{\text{j}}} - {\text{W}}_{{{\text{j1}}}} } \right)^{2} + \left( {{\text{w}}_{{\text{j}}} - {\text{W}}_{{{\text{j2}}}} } \right)^{2} } \right]$$

12 $${\text{w}}_{{\text{j}}} = \frac{{{\text{W}}_{{{\text{j1}}}} \times {\text{W}}_{{{\text{j2}}}} }}{{\mathop \sum \nolimits_{j = 1}^{m} ({\text{W}}_{{{\text{j1}}}} \times {\text{W}}_{{{\text{j2}}}} )}}$$

where, p is the preference coefficient. The results of integrated weights of water quality parameters are given in Table 1.

**Table 1:** Vietnamese groundwater quality standard for water quality parameters used in this study and their relative weights based on the integrated weight calculation.

Parameters	Unit	Vietnamese groundwater quality standard^26^	Weight (w_j_)	Relative weight (W_j_)
pH	–	5.5–7.5	0.034	0.164
Total hardness	mg/L	500	0.099	0.163
NO_3_^−^	mg N/L	15	0.064	0.077
Fe	mg/L	5	0.210	0.098
Pb	mg/L	0.01	0.078	0.119
Hg	mg/L	0.001	0.169	0.136
As	mg/L	0.05	0.163	0.134
Coliforms	MPN/100 mL	3	0.183	0.108
				$$\Sigma {\text{W}}_{{\text{i}}} = 1$$

#### Integrated-weight water quality index (IWQI)

The IWQI values were obtained as follows:

13 $${\text{IWQI }} = \mathop \sum \limits_{{{\text{i}} = 1}}^{{\text{n}}} {\text{W}}_{{\text{j}}} {\text{Q}}_{{\text{j}}}$$

14 $${\text{Q}}_{{\text{j}}} = 100 \times \frac{{{\text{V}}_{{\text{o}}} - {\text{V}}_{{\text{j}}} }}{{{\text{S}}_{{\text{n}}} - {\text{V}}_{{\text{j}}} }}$$

where n is the number of parameters, W_j_ is relative weight for the jth parameter (Table 1), Q_j_: the quality rating of the jth parameter, V_o_: the observed value of jth parameter at a certain monitoring site; V_j_: the ideal values which are considered “0” for drinking water except pH^2,17^. In the case of pH, V_j_ is 7.0 (neutral pH) and S_n_ is 8.5. Vietnamese groundwater quality standard values were used as a reference in this study (Table 1)^26^.

The groundwater quality is categorized into five classes based on the calculation of IWQI: excellent (the values ranged from 0 to 50), good (50 to 100), poor (100 to 200), very poor (200 to 300), and unsuitable for drinking (> 300)^5,16^. Based on the values of IWQI, the spatial distribution of groundwater categories was presented using the using the QGIS version 3.14 software (https://qgis.org/en/site/forusers/download.html). This was based on interpolation with the inverse distance weighted method. The sample points were weighted during interpolation, and the five classes were assigned five different colors.

## Results and discussion

### Hydrochemical and microbiological parameters of groundwater in the Mekong Delta

**Figure 2:**
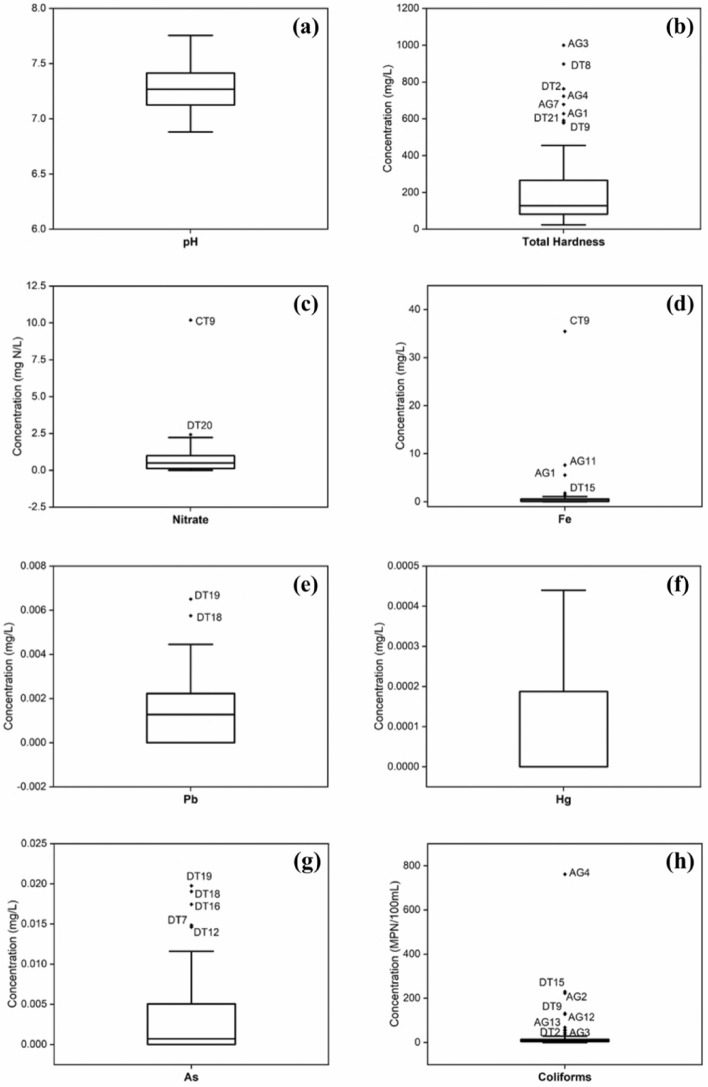
Box and whisker plot of hydrochemical and microbiological parameters in groundwater samples: pH (**a**), total hardness (**b**), nitrate (**c**), Fe (**d**), Pb (**e**), Hg (**f**), As (**g**), and coliforms (**h**). The outliers of the data are presented by the dots with their sampling locations (*AG* An Giang province, *DT* Dong Thap province, *CT* Can Tho city).

The groundwater quality data in the Mekong Delta study area are presented in Table 2 and Fig. 2. The average parameter values were compared with the Vietnamese regulation on groundwater quality QCVN 09-MT:2015/BTNMT^26^. The pH values of the water samples varied from 6.88 to 7.75 with an average of 7.29 ± 0.22 (Fig. 2a), which were still within the Vietnamese standard (5.5–8.5). The pH data observed in this study were similar to the ranges observed in the area by previous studies. During 2009–2018, the average pH value of groundwater samples collected in An Giang province was reported in the range of 6.7–7.2 in the dry season and 6.5–6.9 in the rainy season^13^. pH values in Bac Lieu province were in the range of 7.16–8.20^9^. The difference between the pH variation observed in An Giang and the one observed in Bac Lieu could be attributed to different anthropogenic pollution in both areas since the middle-upper Pleistocene aquifer was highly exploited by industrial and household uses^27^.

**Table 2:** Groundwater quality in An Giang province, Dong Thap province, and Can Tho city in 2019.

Area	Sample	Parameter (unit)							
		pH	Total hardness (mg/L)	NO_3_^–^ (mg/L)	Fe (mg/L)	Pb (× 10^–3^ mg/L)	Hg (× 10^–3^ mg/L)	As (× 10^–3^ mg/L)	Coliforms (MPN/100 mL)
An Giang	AG1	7.25 ± 0.19	627.50 ± 406.59	BDL	5.55 ± 7.85	BDL	BDL	BDL	11.50 ± 16.26
	AG2	7.07 ± 0.03	179.40 ± 0.85	0.08 ± 0.04	0.03 ± 0.05	BDL	BDL	BDL	222.50 ± 293.45
	AG3	7.23 ± 0.53	999.65 ± 390.11	0.11 ± 0.15	0.71 ± 1.00	BDL	BDL	BDL	54.00 ± 55.15
	AG4	7.04 ± 0.23	722.50 ± 45.96	0.02 ± 0.03	BDL	BDL	BDL	BDL	761.50 ± 1044.40
	AG5	7.14 ± 0.03	78.25 ± 11.67	0.07 ± 0.06	0.07 ± 0.10	BDL	BDL	BDL	BDL
	AG6	6.88 ± 0.04	265.00 ± 102.53	1.32 ± 1.53	BDL	BDL	BDL	BDL	29.00 ± 19.80
	AG7	6.91 ± 0.01	678.15 ± 68.09	0.12 ± 0.15	0.03 ± 0.04	BDL	BDL	BDL	11.50 ± 16.26
	AG8	6.94 ± 0.12	396.90 ± 464.00	0.03 ± 0.05	1.45 ± 2.04	BDL	BDL	BDL	45.00 ± 42.43
	AG9	7.14 ± 0.08	581.25 ± 298.75	0.13 ± 0.18	0.51 ± 0.71	BDL	BDL	BDL	26.00 ± 24.04
	AG10	6.93 ± 0.03	366.90 ± 0.85	0.87 ± 1.22	0.92 ± 1.29	BDL	BDL	BDL	11.50 ± 16.26
	AG11	7.08 ± 0.04	260.00 ± 127.28	0.03 ± 0.04	7.63 ± 8.73	BDL	BDL	BDL	37.50 ± 53.03
	AG12	6.99 ± 0.27	151.90 ± 108.75	1.23 ± 1.51	0.13 ± 0.18	BDL	BDL	BDL	127.50 ± 159.10
	AG13	7.00 ± 0.25	195.65 ± 191.84	0.99 ± 1.34	BDL	BDL	BDL	BDL	68.00 ± 35.36
Can Tho city	CT1	7.20 ± 0.00	148.80 ± 128.98	0.05 ± 0.07	0.17 ± 0.15	0.8 ± 1.1	BDL	BDL	2.00 ± 2.83
	CT2	7.38 ± 0.34	103.50 ± 65.76	0.22 ± 0.21	0.23 ± 0.23	2.6 ± 3.7	BDL	0.6 ± 0.8	7.00 ± 0.00
	CT3	7.25 ± 0.14	87.40 ± 46.10	0.15 ± 0.05	0.33 ± 0.15	3.0 ± 4.2	BDL	0.6 ± 0.8	6.50 ± 3.54
	CT4	7.19 ± 0.07	82.90 ± 38.33	0.10 ± 0.07	0.32 ± 0.20	2.0 ± 2.8	BDL	0.8 ± 1.1	7.50 ± 4.95
	CT5	7.26 ± 0.03	104.20 ± 93.06	0.12 ± 0.08	0.18 ± 0.03	1.0 ± 1.4	BDL	1.1 ± 1.6	BDL
	CT6	7.26 ± 0.01	71.20 ± 54.87	0.55 ± 0.77	0.21 ± 0.04	3.1 ± 4.4	BDL	1.1 ± 1.5	5.50 ± 2.12
	CT7	7.30 ± 0.16	68.00 ± 73.54	0.04 ± 0.05	0.66 ± 0.74	1.1 ± 1.6	BDL	BDL	7.00 ± 0.00
	CT8	7.68 ± 0.29	51.00 ± 26.87	0.18 ± 0.25	0.71 ± 0.89	0.7 ± 1.0	BDL	BDL	4.00 ± 0.00
	CT9	7.28 ± 0.39	23.60 ± 2.26	10.19 ± 13.17	35.46 ± 48.85	0.6 ± 0.8	BDL	0.8 ± 1.1	12.00 ± 4.24
	CT10	7.17 ± 0.40	60.80 ± 55.44	0.45 ± 0.52	1.08 ± 1.33	1.2 ± 1.6	BDL	BDL	BDL
	CT11	7.35 ± 0.10	112.00 ± 124.45	0.67 ± 0.40	0.21 ± 0.16	1.3 ± 1.8	BDL	BDL	7.00 ± 0.00
	CT12	7.36 ± 0.06	81.10 ± 83.30	BDL	0.27 ± 0.28	0.8 ± 1.1	BDL	BDL	6.50 ± 3.54
	CT13	7.28 ± 0.18	56.10 ± 47.94	0.05 ± 0.06	0.27 ± 0.18	BDL	BDL	BDL	5.50 ± 2.12
	CT14	7.05 ± 0.51	86.10 ± 90.37	0.02 ± 0.03	0.31 ± 0.18	BDL	BDL	0.7 ± 0.9	4.50 ± 6.36
	CT15	7.73 ± 0.69	103.40 ± 65.90	0.24 ± 0.18	0.28 ± 0.28	BDL	BDL	1.3 ± 1.8	11.50 ± 16.26
	CT16	7.70 ± 0.65	68.50 ± 16.26	0.04 ± 0.05	0.28 ± 0.30	BDL	BDL	0.9 ± 1.2	7.50 ± 4.95
	CT17	7.34 ± 0.11	103.60 ± 65.62	0.27 ± 0.37	0.39 ± 0.48	BDL	BDL	BDL	8.00 ± 1.41
	CT18	7.32 ± 0.05	123.40 ± 122.47	0.03 ± 0.04	0.51 ± 0.50	BDL	BDL	BDL	7.50 ± 10.61
	CT19	7.33 ± 0.04	133.30 ± 136.75	0.73 ± 0.15	0.74 ± 0.85	BDL	BDL	BDL	12.50 ± 12.02
	CT20	7.29 ± 0.05	118.20 ± 115.68	0.43 ± 0.39	0.96 ± 1.16	1.2 ± 1.7	BDL	BDL	5.50 ± 7.78
	CT21	7.16 ± 0.38	85.90 ± 90.65	0.42 ± 0.59	0.64 ± 0.58	1.8 ± 2.5	BDL	BDL	17.00 ± 8.49
	CT22	7.42 ± 0.30	54.00 ± 65.05	0.12 ± 0.17	0.39 ± 0.48	1.3 ± 1.8	BDL	BDL	9.50 ± 7.78
	CT23	7.43 ± 0.30	35.30 ± 20.79	0.24 ± 0.19	0.13 ± 0.10	1.4 ± 2.0	BDL	0.9 ± 1.3	9.50 ± 7.78
	CT24	7.40 ± 0.11	168.50 ± 185.97	0.18 ± 0.14	0.76 ± 0.83	0.9 ± 1.3	BDL	BDL	9.00 ± 2.83
	CT25	7.37 ± 0.16	123.70 ± 122.05	0.36 ± 0.37	0.15 ± 0.05	1.3 ± 1.8	BDL	0.8 ± 1.1	3.50 ± 4.95
	CT26	7.45 ± 0.06	80.90 ± 62.37	0.26 ± 0.01	0.11 ± 0.04	1.3 ± 1.8	BDL	BDL	4.00 ± 0.00
	CT27	7.35 ± 0.18	107.30 ± 102.81	0.43 ± 0.27	0.20 ± 0.13	1.1 ± 1.5	BDL	BDL	3.50 ± 4.95
Dong Thap	DT1	7.20 ± 0.04	160.00 ± 113.14	0.93 ± 0.32	0.08 ± 0.09	1.7 ± 0.1	0.2 ± 0.0	2.7 ± 0.0	11.50 ± 16.26
	DT2	7.37 ± 0.18	762.50 ± 590.43	1.53 ± 0.32	0.06 ± 0.04	1.6 ± 0.2	0.1 ± 0.0	3.0 ± 0.4	21.50 ± 30.41
	DT3	7.02 ± 0.09	454.50 ± 7.78	2.05 ± 0.21	0.16 ± 0.08	2.8 ± 0.7	0.2 ± 0.0	5.5 ± 0.7	58.00 ± 49.50
	DT4	7.10 ± 0.10	220.50 ± 106.77	0.96 ± 0.50	0.09 ± 0.00	2.1 ± 1.1	0.2 ± 0.1	5.5 ± 0.7	BDL
	DT5	6.98 ± 0.07	209.40 ± 92.77	0.78 ± 0.11	0.12 ± 0.02	2.6 ± 1.3	0.2 ± 0.1	4.8 ± 0.7	BDL
	DT6	7.22 ± 0.27	34.75 ± 8.13	0.96 ± 0.50	0.03 ± 0.00	2.3 ± 0.8	0.2 ± 0.0	6.3 ± 0.8	11.50 ± 16.26
	DT7	7.24 ± 0.39	54.50 ± 3.54	1.23 ± 0.74	0.05 ± 0.00	4.5 ± 1.6	0.3 ± 0.0	14.9 ± 0.6	BDL
	DT8	7.12 ± 0.11	897.50 ± 279.31	1.31 ± 0.01	0.17 ± 0.07	1.8 ± 0.8	0.2 ± 0.1	6.6 ± 1.1	11.50 ± 16.26
	DT9	7.30 ± 0.23	577.50 ± 130.81	0.93 ± 0.10	0.03 ± 0.00	1.3 ± 0.3	0.1 ± 0.0	3.4 ± 0.5	131.50 ± 153.44
	DT10	7.41 ± 0.13	209.50 ± 0.71	2.23 ± 0.67	0.74 ± 0.24	1.6 ± 0.4	0.2 ± 0.1	5.0 ± 1.1	21.50 ± 30.41
	DT11	7.18 ± 0.20	385.00 ± 473.76	0.78 ± 0.11	0.04 ± 0.01	2.4 ± 0.9	0.2 ± 0.0	8.0 ± 0.6	BDL
	DT12	7.74 ± 0.51	85.75 ± 6.72	1.13 ± 0.88	0.16 ± 0.02	2.0 ± 0.6	0.2 ± 0.1	14.6 ± 0.9	16.00 ± 22.63
	DT13	7.45 ± 0.02	121.75 ± 40.66	0.96 ± 0.50	1.80 ± 2.36	2.4 ± 0.8	0.2 ± 0.0	6.6 ± 0.6	7.50 ± 10.61
	DT14	7.73 ± 1.06	267.25 ± 235.82	0.91 ± 0.57	0.02 ± 0.01	2.2 ± 0.6	0.2 ± 0.0	4.1 ± 0.5	4.50 ± 6.36
	DT15	7.61 ± 0.95	98.25 ± 61.16	0.73 ± 0.18	1.42 ± 1.99	1.6 ± 0.4	0.2 ± 0.1	5.0 ± 0.4	230.00 ± 325.27
	DT16	7.18 ± 0.92	63.75 ± 44.90	0.83 ± 0.04	0.18 ± 0.22	3.0 ± 0.6	0.4 ± 0.1	17.4 ± 0.5	BDL
	DT17	7.46 ± 0.52	68.75 ± 46.32	2.06 ± 2.06	0.07 ± 0.08	3.0 ± 1.8	0.3 ± 0.0	14.7 ± 0.6	11.50 ± 16.26
	DT18	7.61 ± 0.78	256.00 ± 246.07	0.98 ± 0.17	0.11 ± 0.12	5.8 ± 2.9	0.3 ± 0.0	19.1 ± 0.5	11.50 ± 16.26
	DT19	7.67 ± 0.72	234.00 ± 291.33	1.32 ± 0.59	0.13 ± 0.15	6.5 ± 2.4	0.4 ± 0.0	19.8 ± 0.2	11.50 ± 16.26
	DT20	7.52 ± 0.49	170.00 ± 113.14	2.42 ± 0.30	0.06 ± 0.06	1.7 ± 0.3	0.2 ± 0.1	3.8 ± 1.2	BDL
	DT21	7.76 ± 0.73	591.50 ± 436.28	1.23 ± 0.74	0.02 ± 0.01	1.7 ± 0.3	0.1 ± 0.0	5.3 ± 2.8	11.50 ± 16.26
	DT22	7.12 ± 0.19	390.50 ± 437.70	1.67 ± 1.36	0.08 ± 0.10	3.4 ± 1.8	0.3 ± 0.1	11.6 ± 10.7	11.50 ± 16.26
	DT23	7.05 ± 0.14	135.00 ± 63.64	0.96 ± 0.50	0.04 ± 0.01	2.9 ± 1.8	0.2 ± 0.1	5.0 ± 2.0	BDL
	DT24	7.48 ± 0.51	402.75 ± 434.52	1.45 ± 1.05	0.03 ± 0.01	4.3 ± 4.3	0.3 ± 0.2	11.6 ± 11.1	BDL

*BDL* below detection limit.

Total hardness in groundwater samples indicates high cation concentration, e.g., calcium and magnesium. In this study, the total hardness ranged between 23.6 and 999.7 mg/L, with an average of 231.2 ± 227.4 mg/L (Fig. 2b). Nine out of 64 collected samples (14.1%) exceeded the limit of total hardness for groundwater (500 mg/L). These sampling sites are located in An Giang province (AG1, AG3, AG4, AG7, and AG9 sites) and in Dong Thap province (DT2, DT8, DT9, and DT21 sites). Consumption of these groundwater sources without treatment can lead to health impacts. Comparing with nearby provinces observed within the close time period (2017–2020), the total hardness of groundwater observed in this study was within the range measured in Soc Trang province, which greatly fluctuated from 13 to 3080 mg/L^8^, and higher than the range found in Bac Lieu province, which was from 98 to 172 mg/L^9^.

The NO_3_^−^ concentration in groundwater samples was ranging from below the detection limit (< 0.05 mg/L) to the maximum of 10.19 mg N/L with the average of 0.81 ± 1.34 mg N/L, which all were within 15 mg N/L of the Vietnamese standard (Fig. 2c). Comparable NO_3_^−^ concentrations in groundwater have also been detected by the previously mentioned studies in Mekong delta with the sampling times during 2017–2020: 0.01–2.96 mg N/L in An Giang province^28^, 0.1–260 mg N/L in Soc Trang province^8^, and 0.41–1.91 mg N/L in Bac Lieu province^9^. Since Soc Trang province is located further in the south of this study area (adjacent to Hau Giang province in Fig. 1), and Bac Lieu province is located further in the south next to Soc Trang and Hau Giang provinces, it could be implied that the groundwater in this area of the Mekong Delta has been contaminated with mild NO_3_^−^ pollution during the sampling period. An increase in NO_3_^−^ concentration in groundwater could be influenced by the impacts from domestic and industrial wastewater, agricultural runoff, and excessive fertilizer application^3,17,29^. Previous studies showed various agricultural activities in these provinces such as rice and fruit production^30–32^; therefore, sources of nitrate pollution could be from these agricultural activities in the Mekong Delta area.

Heavy metals in groundwater samples ranged from below the detection limits (< 0.1 mg/L of Fe, < 1 $\times$ 10^–3^ mg/L of As and Pb, and < 3 $\times$ 10^–4^ mg/L of Hg) to 35.5 mg/L of Fe, 6.50 $\times$ 10^–3^ mg/L of Pb, 4.40 $\times$ 10^–4^ of Hg, and 1.98 $\times$ 10^–2^ of As (Fig. 2d–g). The concentrations of these metals detected in groundwater were within the permissible limits except for Fe. Three samples, including AG1, AG11, and CT9, exceeded the limit of Fe in groundwater (5 mg/L) with concentrations of 5.55, 7.63, and 35.46 mg/L, respectively (Fig. 2d). The Fe concentrations observed in An Giang province in this study were much higher than the value observed in Bac Lieu province (1 mg/L), which is located further to the south of Can Tho city^9^. Although the As concentration in this study is within the standard, it was higher than that observed in the nearby Bac Lieu province. Previous studies have reported serious As contamination in An Giang province for over a decade^12,15,33^. With the development of hydropower plant construction in the upper Mekong River and the scarcity of surface water, the increasing use of As-contaminating groundwater is unavoidable^13,34,35^. High concentrations of heavy metal ions in groundwater are normally associated with both natural processes (e.g., water–rock interaction) and human activities (e.g., improper-treated industrial wastewater). The detection of heavy metals in groundwater in this study could also be from the excessive groundwater extraction to serve domestic and irrigation activities, which led to lower water levels and stronger reduction condition that triggers the release of heavy metals into aquifers^36^.

High coliform density was observed in the groundwater samples, which was highest at 761.5 MPN/100 mL with an average value of 33.93 ± 102.53 MPN/100 mL (Fig. 2h). There were 52 groundwater samples (81.3% of total samples) exceeding the standard limit of coliforms (3 MPN/100 mL). It is considered that fecal contamination in groundwater is ubiquitous in the Mekong Delta due to leaking fecal matter from pit latrines, livestock wastewater, and wild animal droppings via improperly protected wells, in which the coliform density can greatly vary from 9 – 9,300 MPN/100 mL^13,28,37^.

### Cluster analysis and principal component analysis of groundwater samples

**Figure 3:**
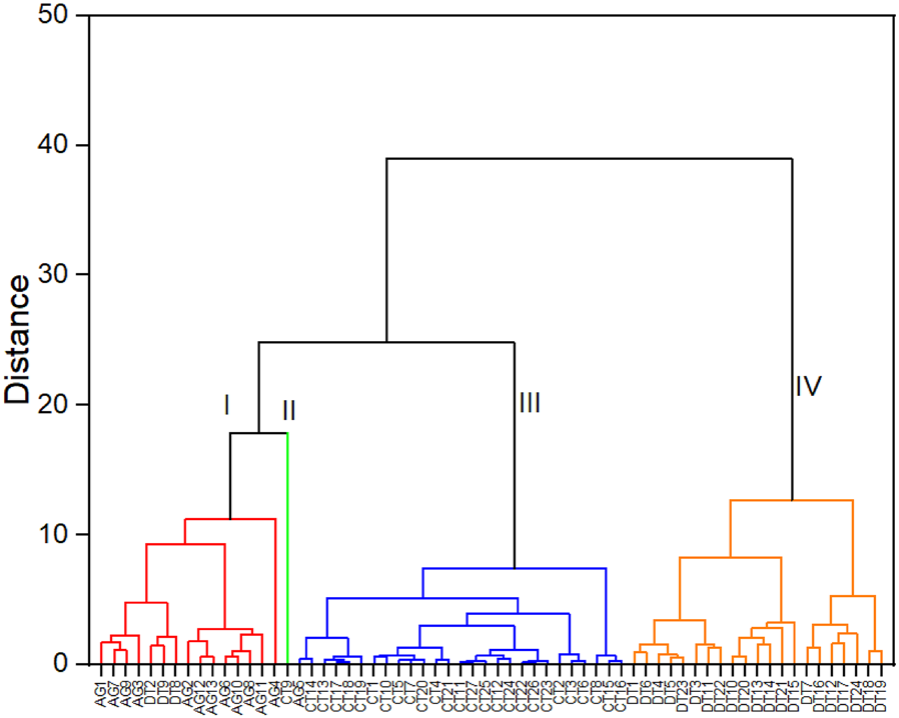
Dendrogram grouping groundwater samples based on hydrochemical and microbiological parameters. Each sample is described on x-axis and Euclid distance is represented on y-axis.

According to CA, 64 groundwater sampling sites were grouped into four clusters based on hydrochemical and microbiological parameters (Euclid distance = 20)^24^ as illustrated in Fig. 3. The groundwater quality characteristics of each cluster are summarized in Table 3. The cluster results also revealed that the groundwater quality in the study area depends on geological locations. Cluster I includes 15 samples (AG1-4, AG6-13, DT2, DT8, DT9), which was highly polluted with coliforms. Groundwater samples in this cluster also showed slight total hardness contamination. However, the hardness of cluster I in Table 3 is a lot higher than other clusters. Most of the samples in Cluster I are located in An Giang province and some in Dong Thap province. CT9 is the only sample classified into Cluster II, which was more polluted with Fe than other clusters. Cluster III includes most of the groundwater samples from Can Tho city from the total of 27 samples (AG5, CT1-8, CT10-27). Lastly, the remaining groundwater samples collected in Dong Thap province (DT1, DT3-7, DT10-24) are grouped into Cluster IV. As same as Cluster I, Cluster II to IV were detected with coliform contamination in groundwater.

**Table 3:** Water quality characteristics of each cluster obtained from hierarchical cluster analysis.

	Cluster I	Cluster II	Cluster III	Cluster IV
pH	7.08	7.28	7.34	7.37
Total hardness	510.82	23.60	92.49	219.69
NO_3_^−^	0.58	10.19	0.24	1.26
Fe	1.15	35.46	0.39	0.26
Pb	3.10 $\times$ 10^–4^	5.50 $\times$ 10^–4^	1.03 $\times$ 10^–3^	2.86 $\times$ 10^–3^
Hg	3.07 $\times$ 10^–5^	0	0	2.34 $\times$ 10^–4^
As	8.60 $\times$ 10^–4^	7.50 $\times$ 10^–4^	3.20 $\times$ 10^–4^	9.10 $\times$ 10^–3^
Coliforms	104.67	12	6.35	19.90

The principal components responsible for the groundwater quality variation in the study area were extracted from the PCA results (Table 4). Three principal components (PC1-3) were extracted based on their eigenvalues higher than 1.0, which could explain 77.2% of the cumulative variation. PC1 accounted for 36.9% of the total variation and was recognized by moderate negative correlation with Pb, Hg, and As. This suggests the pollution from water–rock interactions and anthropogenic sources like industrial wastewater and landfill leachate, which leads to the dissolution of metallic compounds into groundwater^9,13^. Can Tho city is the largest city in the Mekong Delta and is well-known for its commercial, cultural, and industrialized activities while small-scale industries such as craft production can be found in Dong Thap province, leading to the contamination of heavy metals in the surface water and soil that can be leached to groundwater^38,39^. PC2 was associated with a moderate positive correlation with NO_3_^−^ and Fe, accounting for 23.0% of the total variation. Anthropogenic sources such as excessive fertilizer use, industrial and domestic wastewater, agricultural runoff, and aquacultural wastewater are the sources of NO_3_^−^ pollution^3,17,29^. Rice production has been reported as the dominant agricultural activity in An Giang province^30^ while rice starch production, fish pond, and livestock farms could be found in Dong Thap province^39^. These activities could then contribute to the presence of NO_3_^−^ in groundwater of the study area. Since Fe can act as an electron donor in the denitrification process^40^, high Fe concentration in the groundwater samples can possibly reduce NO_3_^−^ concentration. The third principal component, PC3, could explain 17.3% of the total variation and was characterized by a moderate positive correlation with total hardness and coliforms. Industrial wastes and natural hydrogeochemical activities are primary sources of calcium and magnesium cations^41^, which significantly contribute to the total hardness of groundwater. Moreover, according to the analysis, the groundwater in the study area is also susceptible to pathogenic contamination. High coliform density is associated with leaking contaminants from fecal sources such as pit latrines, sewage pipes, or livestock wastes from the farms in the study area^13,28,37,39^.

**Table 4:** Loading values of water quality parameters in each principal component.

Variable	PC1	PC2	PC3	PC4	PC5	PC6	PC7	PC8
pH	− 0.263	− 0.002	− 0.377	− 0.759	0.459	− 0.017	0.033	0.011
Total hardness	0.054	− 0.140	**0.670**	0.077	0.706	− 0.142	− 0.036	− 0.045
NO_3_^−^	− 0.167	**0.670**	0.169	0.002	0.013	0.065	0.642	− 0.279
Fe	0.033	**0.712**	0.085	− 0.060	0.027	− 0.117	− 0.628	0.270
Pb	− **0.529**	− 0.075	0.010	0.102	− 0.159	− 0.823	0.023	− 0.004
Hg	− **0.543**	− 0.069	0.204	0.067	− 0.046	0.375	0.143	0.701
As	− **0.550**	− 0.061	0.139	0.033	− 0.089	0.374	− 0.412	− 0.597
Coliforms	0.147	− 0.102	**0.559**	− 0.631	− 0.504	− 0.059	0.015	− 0.002
Eigenvalues	2.96	1.84	1.38	0.8	0.65	0.2	0.11	0.05
%Variation	36.9	23.0	17.3	10.0	8.1	2.5	1.4	0.7
Cum.%Variation	36.9	59.9	77.2	87.2	95.4	97.9	99.3	100

The numbers in bold indicate a strong correlation between the variables and PCs.

### Integrated-weight groundwater quality index for groundwater classification

The results of IWQI calculation and groundwater quality classification for drinking purposes are presented in Table 5. The IWQI values varied from 4 to 2761 with a mean of 139 ± 370. Out of 64 groundwater samples, over half (53.1%) were classified as excellent for human consumption. There were 16 (25%), 6 (9.4%), and 3 (4.7%) samples categorized as good, poor, and very poor water quality for drinking purposes, respectively. In addition, 5 samples including AG2, AG4, AG12, DT9, and DT15 had the IWQI values over 300, which were considered unsuitable for any drinking purposes. One of the reasons for groundwater having inadequate quality for human consumption is high coliform density. The previous CA results showed that all clusters were contaminated with coliforms. This was also proved by the IWQI results, which showed a high positive correlation coefficient (0.99) with coliform (Table 6). Even though As, Hg, and Pb were highly correlated with each other, these parameters were within the allowable limits. Besides, the remaining parameters have no correlation or very weak correlation with each other. Therefore, it might be deduced that changes in groundwater quality in the study area were significantly attributable to coliform density.

**Table 5:** IWQI values of sample sites and corresponding groundwater categories (Rank I: excellent, Rank II: good, Rank III: poor, Rank IV: very poor, and Rank V: unsuitable for drinking).

Samples	IWQI	Rank	Samples	IWQI	Rank	Samples	IWQI	Rank
AG1	75	II	CT10	8	I	DT5	14	I
AG2	806	V	CT11	35	I	DT6	52	II
AG3	231	IV	CT12	31	I	DT7	18	I
AG4	2761	V	CT13	25	I	DT8	80	II
AG5	4	I	CT14	20	I	DT9	499	V
AG6	112	III	CT15	54	II	DT10	97	II
AG7	63	II	CT16	38	I	DT11	23	I
AG8	177	III	CT17	37	I	DT12	79	II
AG9	115	III	CT18	35	I	DT13	48	I
AG10	55	II	CT19	55	II	DT14	40	I
AG11	159	III	CT20	30	I	DT15	846	V
AG12	464	V	CT21	69	II	DT16	18	I
AG13	251	IV	CT22	43	I	DT17	61	II
CT1	16	I	CT23	42	I	DT18	74	II
CT2	37	I	CT24	45	I	DT19	76	II
CT3	33	I	CT25	23	I	DT20	18	I
CT4	35	I	CT26	24	I	DT21	75	II
CT5	8	I	CT27	22	I	DT22	67	II
CT6	30	I	DT1	54	II	DT23	13	I
CT7	33	I	DT2	111	III	DT24	31	I
CT8	26	I	DT3	232	IV			
CT9	123	III	DT4	15	I

**Table 6:** Correlation matrix between water quality parameters and overall IWQI values.

		pH	Hardness	NO_3_^−^	Fe	Pb	Hg	As	Coliform	IWQI
pH	Correlation coefficient	1								
	p-values	–								
Hardness	Correlation coefficient	− 0.22	1							
	p-values	0.07	–							
NO_3_^−^	Correlation coefficient	0.04	− 0.04	1						
	p-values	0.73	0.75	–						
Fe	Correlation coefficient	− 0.03	− 0.09	0.83	1					
	p-values	0.81	0.50	0.00	–					
Pb	Correlation coefficient	0.30	− 0.10	0.16	− 0.14	1				
	p-values	0.01	0.43	0.20	0.27	–				
Hg	Correlation coefficient	0.26	0.09	0.24	0.13	0.81	1			
	p-values	0.04	0.48	0.06	0.30	0.00	–			
As	Correlation coefficient	0.31	0.01	0.21	− 0.11	0.82	0.93	1		
	p-values	0.01	0.94	0.09	0.40	0.00	0.00	–		
Coliform	Correlation coefficient	− 0.17	0.30	− 0.07	− 0.03	− 0.20	− 0.09	− 0.11	1	
	p-values	0.18	0.02	0.57	0.80	0.12	0.49	0.38	–	
IWQI	Correlation coefficient	− 0.17	0.32	− 0.05	− 0.01	− 0.19	− 0.08	− 0.10	0.99	1
	p-values	0.19	0.01	0.70	0.94	0.13	0.55	0.43	0.00	–

**Figure 4:**
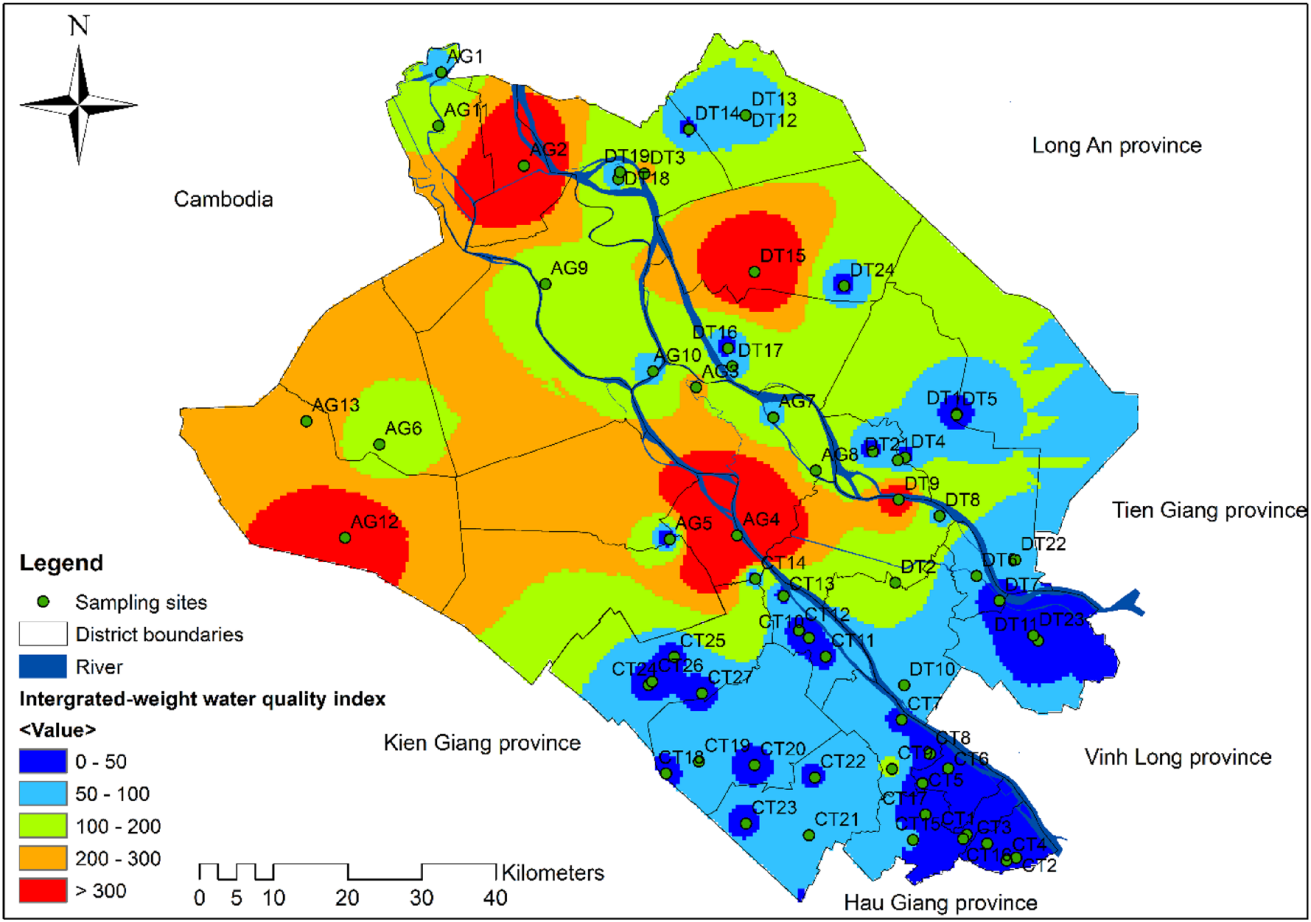
Spatial distribution map of groundwater classification for drinking purposes based on the IWQI values. The map was generated by the software QGIS version 3.14 (https://qgis.org/en/site/forusers/download.html) licensed under GNU General Public License (CC BY-SA 3.0).

Based on the interpolation of IWQI values with inverse distance weighting, the distribution map of groundwater quality categories for drinking purposes were constructed (Fig. 4). It can be seen that most of the samples classified unsuitable for drinking (AG2, DT15, AG4, DT9) are located near large rivers. This could imply that the groundwater quality in these areas depends on the surface water quality because contaminants can directly infiltrate into the aquifers. Therefore, agricultural runoff and the discharge of untreated or improperly treated industrial, domestic, and aquacultural wastewater into surface water could consequently contaminate the groundwater in the region. It was found that surface water and groundwater in An Giang province was heavily polluted with *Escherichia coli* and coliforms due to the emergence of burial swine pits in 2019^28^; thus, the leachate containing pathogens from these sites could leak into surrounding water bodies. In this study, the majority of groundwater samples with lower IWQI values were recorded in the east and southeast parts of the study area where these sampling points are located far from the burial pits. It has been shown that the fine-grained sediments separating the water table from the vadose zone can reduce the transport of microorganisms into groundwater^42^, which could prevent the transfer of *E. coli* and coliforms from the burial pits to the sampling locations. However, since microorganisms may not be degraded by this process, the continuous flow of water in the subsurface will eventually transport them to the area^43^. Therefore, lower IWQI values observed in An Giang area could be the result of slow transport of coliforms. From the findings, it can be implied that groundwater quality in this area was continuously affected by human activities, and it cannot be used as a safe freshwater resource for human consumption.

## Conclusion

This study evaluated groundwater quality in the Mekong Delta of Vietnam using multivariate statistical methods (CA and PCA) and IWQI. The results showed that the values of pH, NO_3_^−^, Hg, As, and Pb in 64 sampling sites were within the Vietnamese standard. Meanwhile, total hardness and Fe concentrations in some sites exceeded the groundwater quality standard, and high coliform contamination was detected at over 80% of total samples. Sampling sites were classified into 4 clusters according to the differences in water quality parameters and geological characteristics. The results of PCA revealed that 3 PCs could explain 77.2% of the total variation of groundwater quality in this study. PC1 showed moderate correlation with Pb, Hg, and As while PC2 and PC3 had moderate correlation with NO_3_^−^ and Fe, and total hardness and coliforms, respectively. Even though a very strong correlation was observed between IWQI values and coliforms, more than 50% of the sampling sites still contain groundwater with excellent quality for drinking purposes. The results of this study have partially evaluated the status of groundwater quality in the Mekong Delta area that can support policymakers in developing future strategies for water treatment and water resource management.

## Data Availability

The datasets generated during and/or analyzed during the current study are available from the corresponding author on reasonable request.
